# Epilepsy Occurrence and Circadian Rhythm: A Bibliometrics Study and Visualization Analysis via CiteSpace

**DOI:** 10.3389/fneur.2020.00984

**Published:** 2020-11-05

**Authors:** Dongling Zhong, Shanxia Luo, Linli Zheng, Yonggang Zhang, Rongjiang Jin

**Affiliations:** ^1^Department of Periodical Press and National Clinical Research Center for Geriatrics, West China Hospital, Sichuan University, Chengdu, China; ^2^School of Health Preservation and Rehabilitation, Chengdu University of Traditional Chinese Medicine, Chengdu, China; ^3^Mental Health Center, West China University Hospital, Sichuan University, Chengdu, China

**Keywords:** epilepsy, circadian rhythm, bibliometrics, visualization analysis, CiteSpace, review

## Abstract

**Objective:** This study aimed to review the research status and to demonstrate the hot spots and frontiers of epilepsy and circadian rhythm via CiteSpace.

**Method:** We searched Web of Science (WoS) for studies related to epilepsy and circadian rhythm from inception to 2020. CiteSpace was used to generate network maps about the collaborations between authors, countries, and institutions and reveal hot spots and frontiers of epilepsy and circadian rhythm.

**Results:** A total of 704 studies related to epilepsy and circadian rhythm from the WoS were retrieved. Sanchez-Vazquez FJ was the most prolific author (17 articles). The USA and University of Murcia were the leading country and institution in this field with 219 and 22 publications, respectively. There were active collaborations among the authors, countries, and institutions. Hot topics focused on the interaction between epilepsy and circadian rhythm, as well as possible novel treatments.

**Conclusions:** Based on the results of CiteSpace, the current study suggested active cooperation between authors, countries, and institutions. Major ongoing research trends include the circadian rhythm of epilepsy based on different epileptic focus and the interaction between epilepsy and circadian rhythm, especially through melatonin, sleep–wake cycles, and clock genes, which may implicate possible treatments (such as chronotherapy, neural stem cells transplantation) for epilepsy in the future.

## Background

Epilepsy is a common neurological disease with an unpredictable occurrence caused by abnormal discharge of brain neurons with high incidence and high cost (1, 2). A systematic review estimated that the point prevalence of active epilepsy was 6.38%0, while the lifetime prevalence was 7.6%0, especially higher in low- to middle-income countries (3). In the People's Republic of China, the lifetime epilepsy prevalence had increased to 4.57%0 in the 0–4 year group and 8.43%0 in the 30–34 year group by 2015 (4). Epilepsy affects about 65 million people in the entire world (5) and not only brings heavy economic burden to families and societies but also results in psychological problems (6).

The unpredictable nature of epileptic seizures makes it difficult to diagnose and treat. Current evidence and technology like electroencephalogram (EEG) recordings help confirm the association between epilepsy and circadian rhythm (7, 8), which may provide a new basis for the prediction and treatment of epilepsy. The circadian rhythm refers to the sleep–wake cycles, physiological and psychological behavior, and biology under the control of the circadian clock, including changes in sleep and wakefulness, core body temperature, blood pressure, and hormone levels. Evidence showed that circadian rhythm changed in epilepsy subjects (8), including hormones, body temperature, activity, and sleep–wake cycles (9, 10, 37). A better understanding of the relationship between epilepsy occurrence and circadian rhythm may provide more treatment approaches such as chronotherapy to control epilepsy. Therefore, visualizing the research status, hot spots, and frontiers of epilepsy and circadian rhythm is meaningful and necessary. CiteSpace is an application for analyzing and visualizing trends and patterns in scientific literature with metrology, co-occurrence analysis and cluster analysis based on Java (11, 12). By means of CiteSpace, our study focused on the network of co-authors, countries, and institutions; co-cited references analysis; co-occurring keywords and cluster analysis; and the burst of keywords and explored the hot spots and trends of epilepsy and circadian rhythm.

## Method

### Search Strategy

We retrieved related studies using the following terms: epilepsy and circadian rhythm. The index used was the Science Citation Index Expanded of Web of Science (SCI-EXPANDED), and the time span was from inception to 2020. A detailed search strategy was reported in Box 1.

BOX 1 **Search strategy.**

#1 (Epilepsy*) OR (Seizure Disorder*) OR (Aura*)

#2 (Circadian Rhythm*) OR (Diurnal Rhythm* OR Twenty* Four Hour Rhythm*

#1 AND #2

Index = the Science Citation Index Expanded of Web of Science (SCI-EXPANDED)

Time Span = inception−2020

### Visualization Analysis Tool—CiteSpace

**Figure 1:**
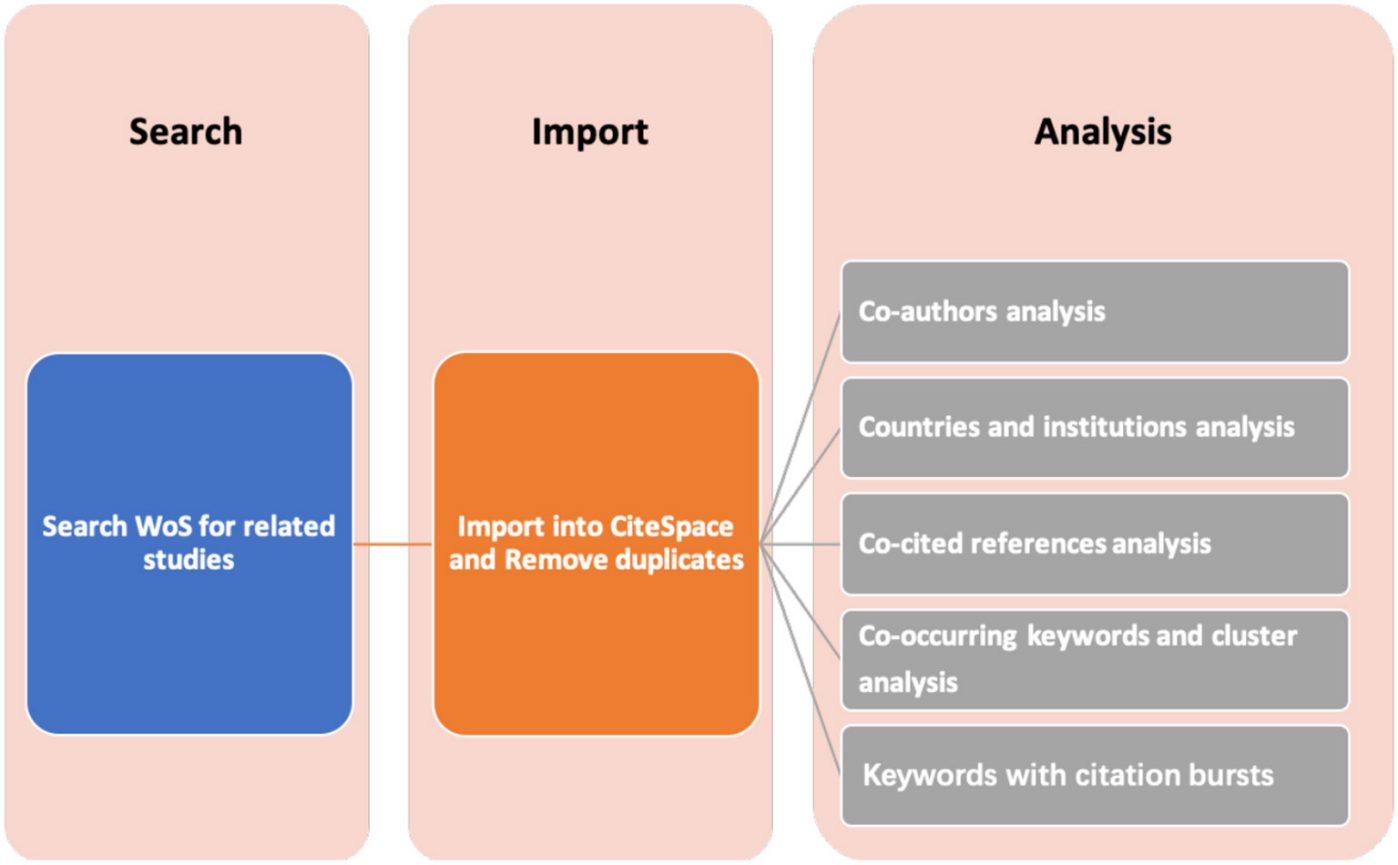
Flow chart for CiteSpace.

CiteSpace was used for bibliometrics and visualization analysis, which can provide critical points in the development of epilepsy and circadian rhythm, including the fast-growth topical areas and citation hotspots. CiteSpace contains three central concepts, including burst detection, betweenness centrality, and heterogeneous networks, which help to identify the nature of a research front, label a specialty, and detect emerging trends and abrupt changes in a timely manner (12). There are various nodes and links in different CiteSpace visualization knowledge maps; nodes with high centrality are usually recognized as hot spots or turning points in a field. Data from WoS were exported in plain text format with full records and references, named “download_XXX.txt,” and then were imported into CiteSpace 5.3.R4 for analysis (Figure 1). Relevant visual maps were drawn and interpreted.

## Results

### Publication Years

**Figure 2:**
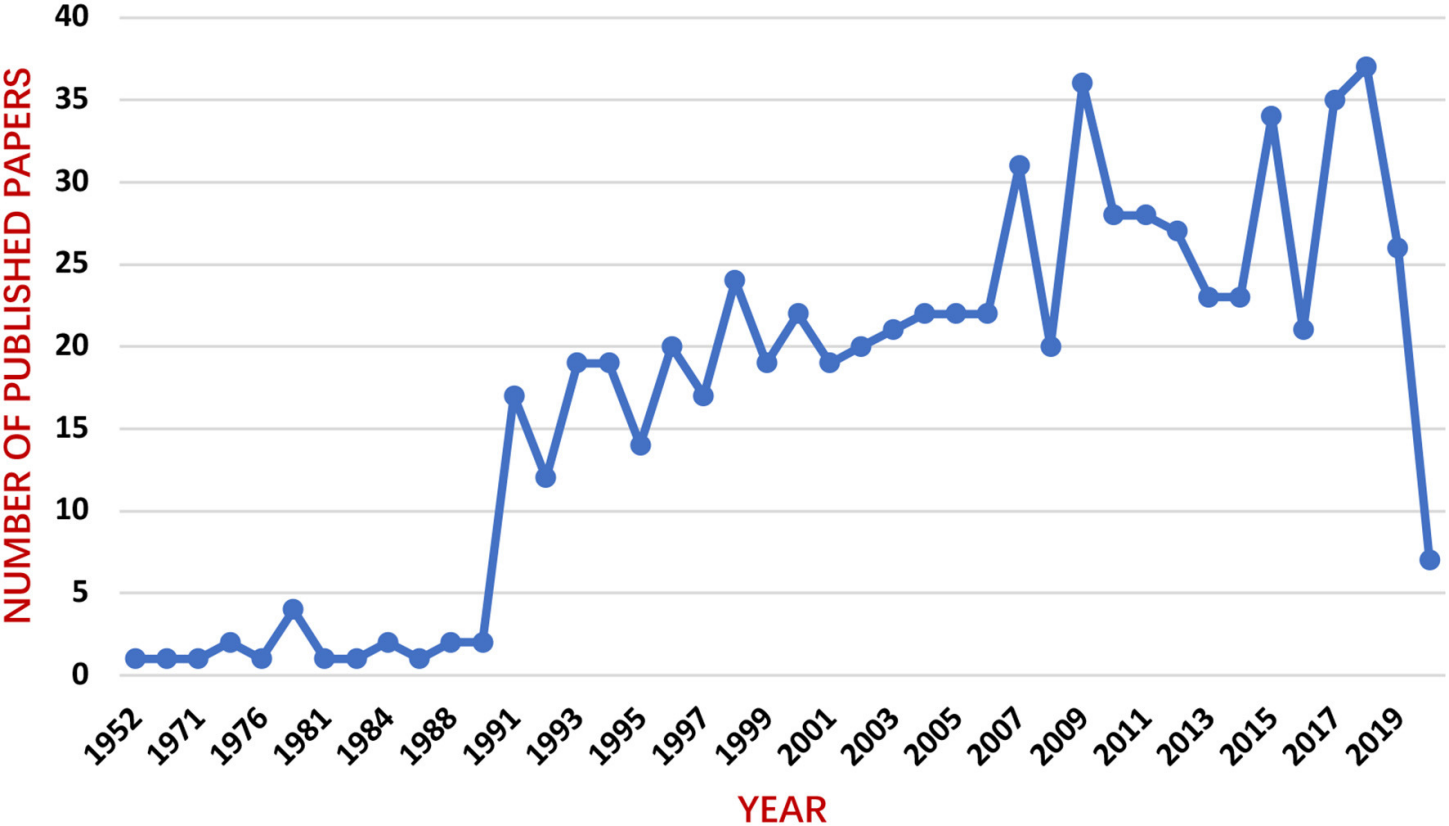
Annual trend chart of publications.

A total of 704 relevant papers were obtained after removing two duplicates (publications in 2020 were not fully included). As shown Figure 2, the number of publications about epilepsy and circadian rhythm has generally steadily increased but with some fluctuations, especially growing rapidly in 1990s, which indicates that this field received great attention in the 1990s.

### Co-authors Analysis

**Figure 3:**
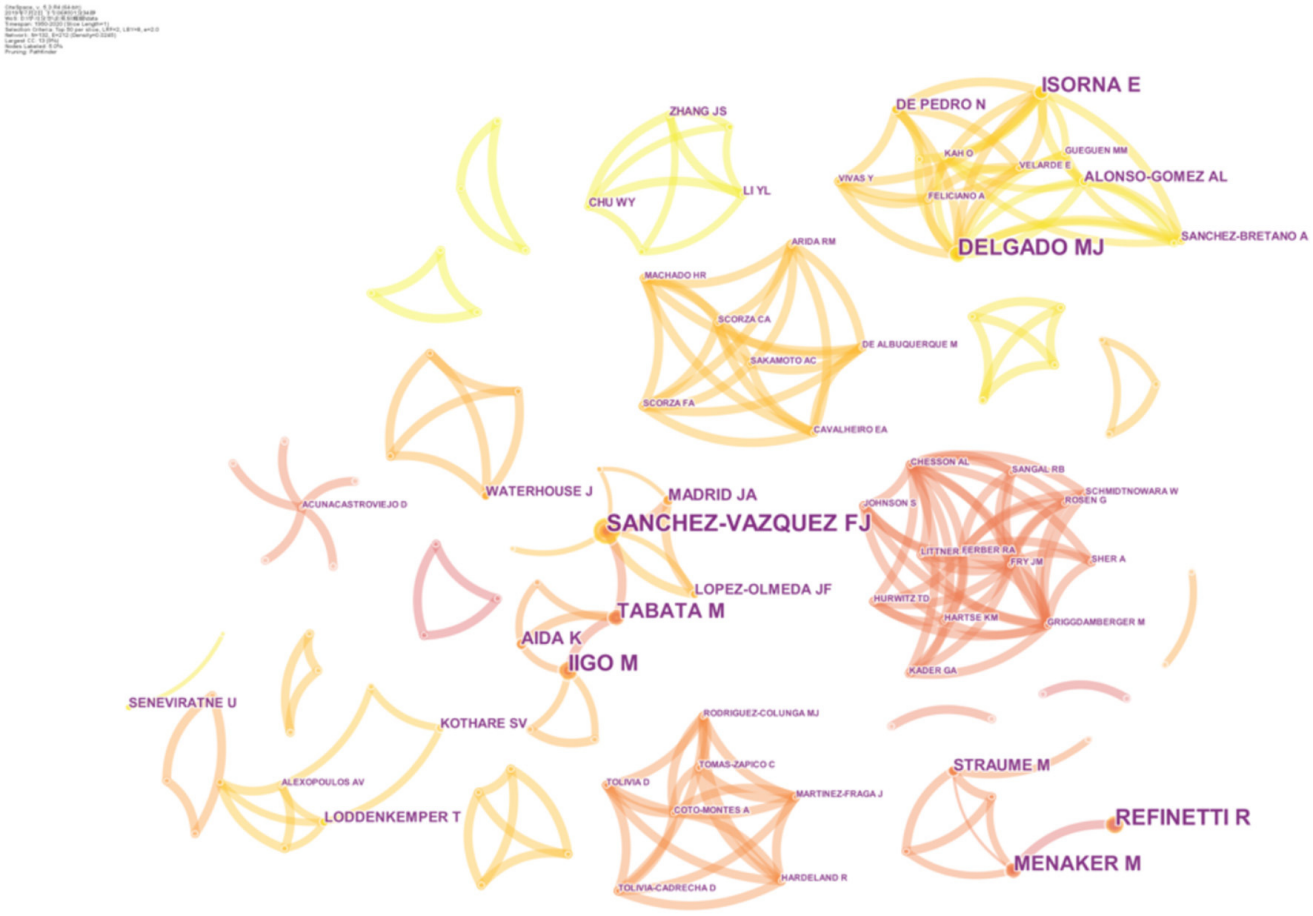
The network of co-authors.

In the network of co-authors, each node represents one author, and the size of the node indicates the number of publications of the author. A larger node means more publications. The top 10 authors contributed 109 articles (15.48%) (Table 1). The most productive author was SANCHEZ-VAZQUEZ FJ (17 articles, 2.41%), followed by IIGO M (14 articles, 1.99%), REFINETTI R (14 articles, 1.99%), DELGADO MJ (13 articles, 1.85%), MENAKER M (11 articles, 1.56%), and TABATA M (11 articles, 1.56%). The lines between different authors represent collaboration. As presented in Figure 3, prolific authors often have stable collaboration with other authors.

**Table 1:** The top 10 authors of epilepsy and circadian rhythm.

**Rank**	**Author**	***N* (%)**	**Year**
1	SANCHEZ-VAZQUEZ FJ	17 (2.41%)	1998
2	IIGO M	14 (1.99%)	1996
3	REFINETTI R	14 (1.99%)	1992
4	DELGADO MJ	13 (1.85%)	2010
5	MENAKER M	11 (1.56%)	1992
6	TABATA M	11 (1.56%)	1996
7	ISORNA E	10 (1.42%)	2010
8	AIDA K	7 (0.99%)	1997
9	MADRID JA	6 (8.52%)	2001
10	STRAUME M	6 (8.52%)	1998

### Countries and Institutions Analysis

**Figure 4:**
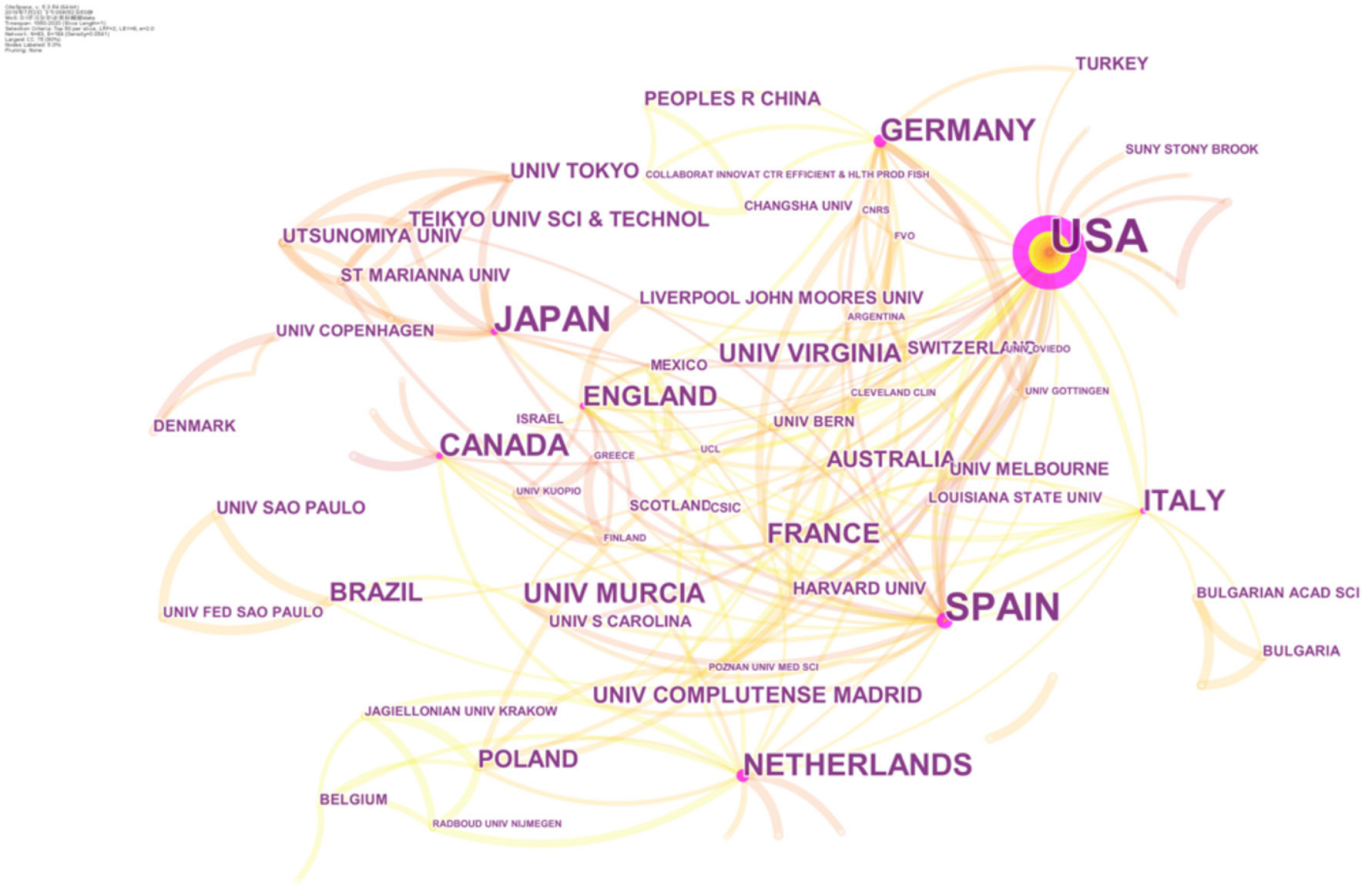
The network of countries and institutions.

The top 10 countries and top 5 institutions contributed 527 (74.86%) and 67 (9.52%) articles, respectively (Table 2). The top 5 countries were the USA, Spain, Japan, Germany, and Netherlands. And the top five institutions were the University of Murcia, University of Virginia, University Complutense Madrid, University of Tokyo, and Teikyo University of Science & Technology. Results showed that countries and institutions actively collaborated, especially with the USA and European countries (Figure 4).

**Table 2:** The top 10 countries and top 5 institutions of epilepsy and circadian rhythm.

**Rank**	**Country**	***N* (%)**	**Institution**	***N* (%)**
1	USA	219 (31.11%)	University of Murcia	22 (3.13%)
2	Spain	89 (12.64%)	University of Virginia	15 (2.13%)
3	Japan	50 (7.10%)	University Complutense Madrid	12 (1.70%)
4	Germany	32 (4.55%)	University of Tokyo	9 (1.28%)
5	Netherlands	29 (4.12%)	Teikyo University of Science & Technology	9 (1.28%)
6	Canada	28 (3.98%)		
7	Italy	23 (3.27%)		
8	France	22 (3.13%)		
9	England	20 (2.84%)		
10	Brazil	15 (2.13%)		

### Co-cited References Analysis

Table 3 presented the top 10 co-cited references related to epilepsy and circadian rhythm, and they were co-cited more than 160 times. The first co-cited reference was an article published by Ralph et al., (13) which proved the pacemaker role of the suprachiasmatic nucleus in a mammalian circadian system and laid a foundation for future research. The second co-cited reference was published by Durazzo et al., (14) and results of this article suggested that some epilepsy does not occur randomly. Endogenous circadian rhythms and rhythmic exogenous factors may play substantial roles in seizure occurrence. The third co-cited reference explored the day/night patterns of focal seizures (15). This article showed that both sleep/wake state and day/night or circadian rhythms may affect seizure proclivity, with different effects depending on the location of the epileptogenic region.

**Table 3:** The top 10 co-cited references of epilepsy and circadian rhythm.

**Rank**	**Co-cited reference**	**Impact factor**	**Co-citation**
1	Ralph MR, 1990, Science, V247, P975, Doi 10.1126/Science.2305266	41.037	22
2	Durazzo TS, 2008, Neurology, V70, P1265, Doi 10.1212/01.Wnl.0000308938.84918.3f	8.689	21
3	Pavlova MK, 2004, Epilepsy Behav, V5, P44, Doi 10.1016/J.Yebeh.2003.10.013	2.378	19
4	Feliciano A, 2011, J Biol Rhythm, V26, P24, Doi 10.1177/0748730410388600	2.473	17
5	Velarde E, 2009, J Biol Rhythm, V24, P104, Doi 10.1177/0748730408329901	2.473	16
6	Hofstra WA, 2009, Epilepsia, V50, P2019, Doi 10.1111/J.1528-1167.2009.02044.X	5.562	16
7	Loddenkemper T, 2011, Neurology, V76, P145, Doi 10.1212/Wnl.0b013e318206ca46	8.689	15
8	Hofstra WA, 2009, Epilepsy Behav, V14, P617, Doi 10.1016/J.Yebeh.2009.01.020	2.378	13
9	Dibner C, 2010, Annu Rev Physiol, V72, P517, Doi 10.1146/Annurev-Physiol-021909-135821	17.902	13
10	Vera LM, 2013, Chronobiol Int, V30, P649, Doi 10.3109/07420528.2013.775143	2.562	12

### Co-occurring Keywords and Cluster Analysis

**Figure 5:**
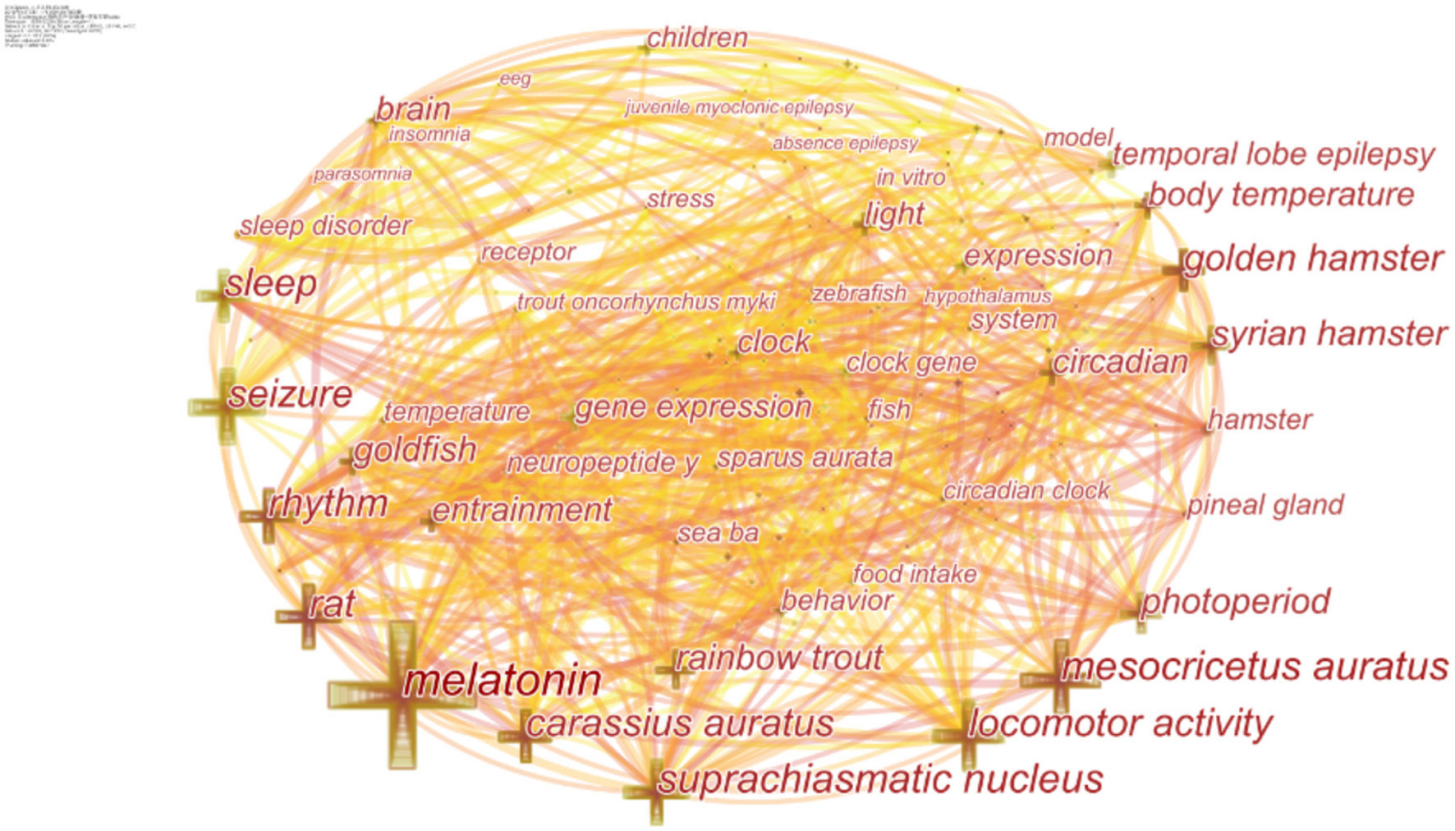
Co-occurring keywords map.

High-frequency keywords represent a hot topic in a research field, while high-centrality keywords reflect the position and influence of the corresponding research content in this research field. As shown in Table 4, hot keywords in the frequency and centrality order were melatonin (MT) (frequency: 142, centrality: 0.16), mesocricetus auratus (frequency: 84, centrality: 0.14), and carassius auratus (frequency: 66, centrality: 0.11). Other keywords included gene expression, temporal lobe epilepsy, neuropeptide, pineal gland, children, and so on (Figure 5).

**Table 4:** Top 10 keywords in terms of frequency and centrality of epilepsy and circadian rhythm.

**Rank**	**Frequency**	**Keywords**	**Centrality**	**Keywords**
1	321	Circadian rhythm	0.2	Epilepsy
2	142	Melatonin	0.16	Melatonin
3	135	Epilepsy	0.15	Entrainment
4	84	Mesocricetus auratus	0.14	Circadian rhythm
5	76	Suprachiasmatic nucleus	0.14	Mesocricetus auratus
6	72	Seizure	0.13	Brain
7	72	Locomotor activity	0.12	Circadian
8	69	Sleep	0.11	Carassius auratus
9	67	Rat	0.1	Golden hamster
10	66	Carassius auratus	0.1	Rainbow trout

**Figure 6:**
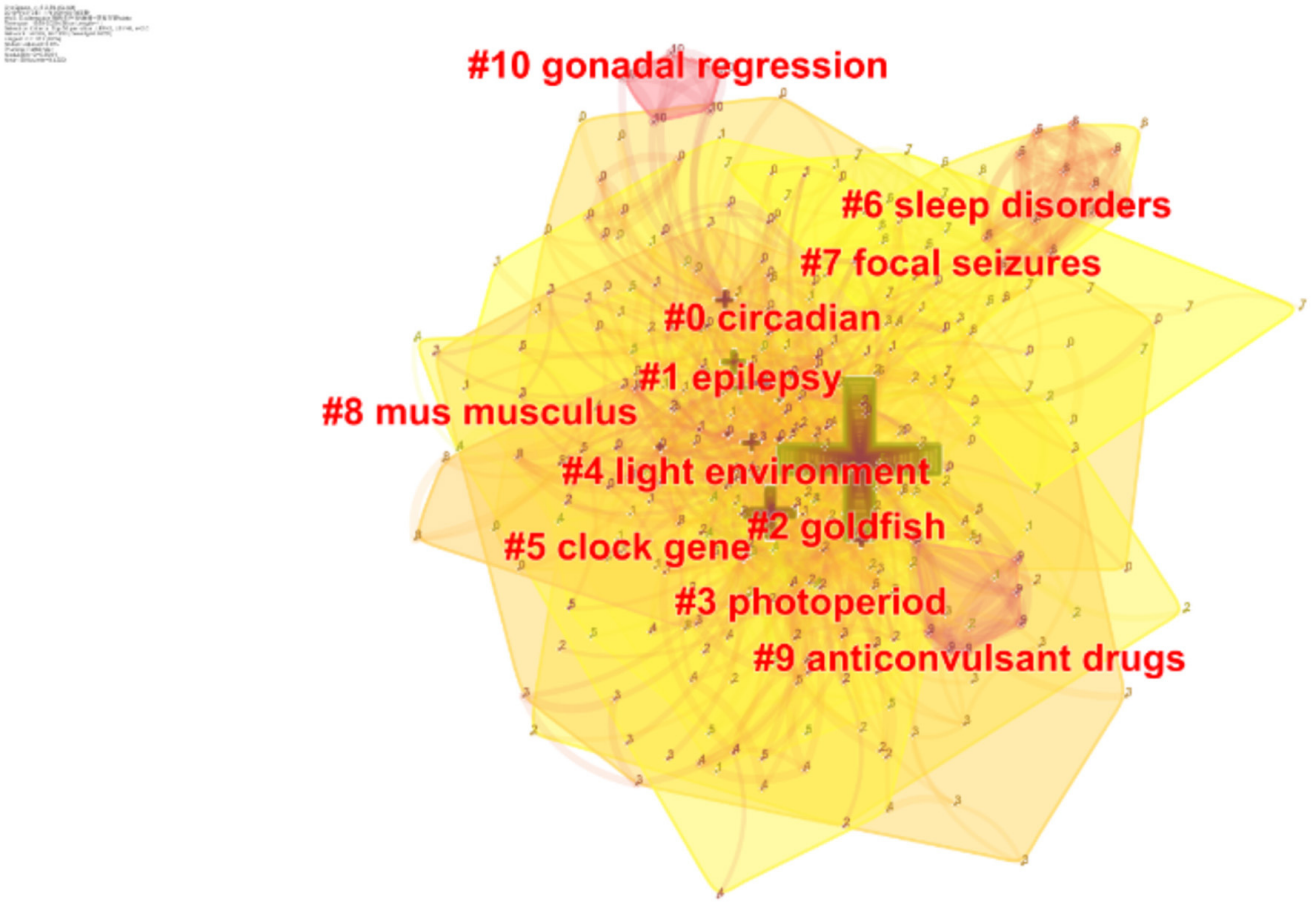
Keywords cluster analysis co-occurrence map.

Clustering analysis revealing the main topics was performed for co-occurrence keywords using CiteSpace. The mean silhouette is usually used for evaluating the clusters. In general, a silhouette value over 0.7 means the cluster is high in efficiency and convincing; if it is above 0.5, the cluster is generally considered reasonable. Eventually, we obtained 10 clusters, and the silhouette value for each cluster was over 0.5, suggesting that the results were reliable and meaningful. Among them, seven clusters had a silhouette value over 0.7 (Figure 6, Table 5).

**Table 5:** Keywords cluster analysis (the silhouette value is over 0.7).

**Cluster ID**	**Silhouette**	**Label (LLR)**	**Included keywords (top 15)**	**Cite year**
4	0.785	Light environment	Stress; innate immunity; light–darkness cycle; pikeperch; otolith; light spectrum; pike perch; circadian axis; hpi axis; ketone body regulation; neurological disorders; epigenetics; immunity; ketogenic diet; metabolic disorders	2007
5	0.701	Clock gene	Melatonin; fasting; liver; dopamine receptors; pituitary; muscle functional gene; intestine; circadian system; dopamine; annual reproductive cycle; food hoarding; photoperiodism; green wavelength; refractoriness; functional genes	2006
6	0.867	Sleep disorders	Insomnia; polysomnography; circadian rhythm disorders; parasomnias; narcolepsy; practice guidelines; practice parameters; standards of practice; sleep apnea syndrome; periodic limb movement disorder; restless legs syndrome; disease symptoms; neurologic diseases; international classification of sleep disorders; interictal	2004
7	0.848	Focal seizures	Polyspike; spike-wave; photoparoxysmal response; absence epilepsy; spike-wave discharges; absence; wag; myoclonus; rij rats; tonic–clonic seizure; genetic model; juvenile myoclonic epilepsy; rem sleep; wag/rij rats; sleep-related epilepsy	2013
8	0.929	Mus musculus	Dark adaptation; photic entrainment; period length; interindividual variability; stability; phase response; circadian rhythm; running wheel; arvicanthis niloticus; dark pulses; light pulses; mongolian gerbils; locomotor activity; mesocricetus auratus; masking	2001
9	0.975	Anticonvulsant drugs	Experimental seizures; electroshock; pentylenetetrazol; epilepsy; melatonin analogs; sedative; anticonvulsant; gamma distribution; rodents; anxiolytic; automated seizure detection; preclinical research; sleep disturbance; rett syndrome; seizure	1991
10	0.983	Gonadal regression	Short photoperiod; long photoperiod; recrudescence; golden hamster; circadian rhythms; circadian rhythm; epilepsy; melatonin; locomotor activity; circadian; photoperiod; goldfish; mesocricetus auratus; suprachiasmatic nucleus; sleep disorders	1991

### Keywords With Citation Bursts

**Figure 7:**
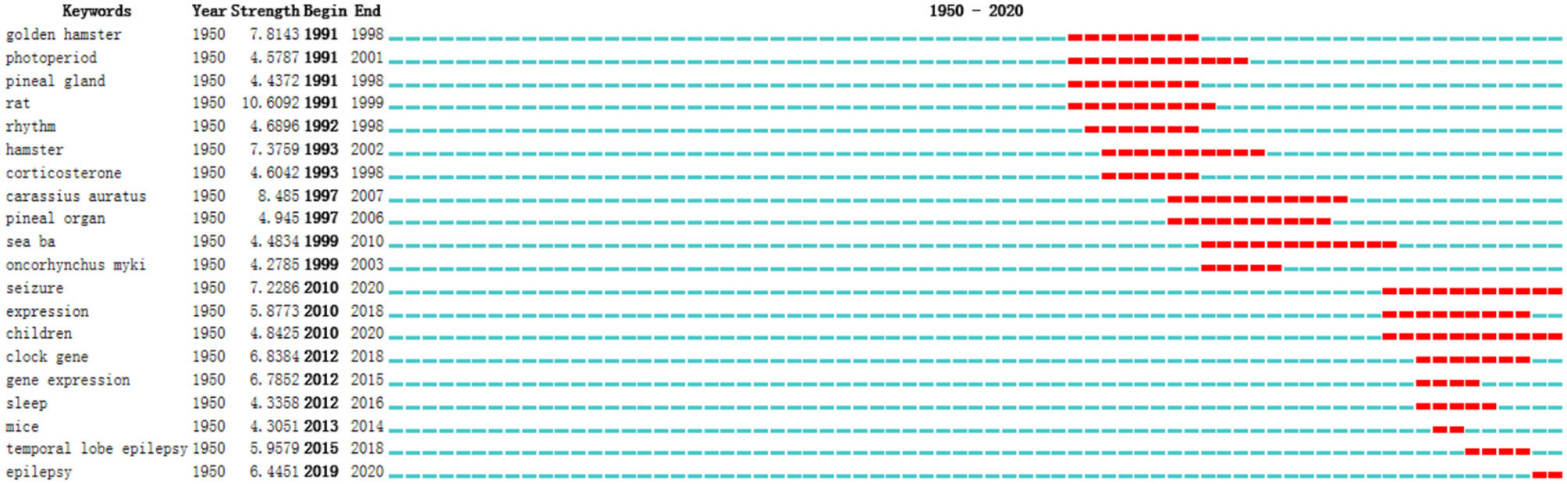
Top 20 keywords with the strongest citation bursts.

Figure 7 showed the top 20 keywords with the strongest citation bursts. The blue line represents the time interval, while the red line indicates the time period in which a keyword was found to have a burst (16). Keywords with citation bursts first appeared in 1950 (golden hamster, photoperiod, and pineal gland), along with the strongest keyword (rat). The most recent keyword with citation bursts appeared in 2019 (epilepsy). And 3 keywords with citation bursts continue to 2020 (seizure, children, and epilepsy).

## Discussion

### Summary of Findings

The current study aimed to review the research status and demonstrate the hot spots and frontiers of epilepsy and circadian rhythm via CiteSpace. A total of 704 studies related to epilepsy and circadian rhythm were retrieved from the WoS. Sanchez-Vazquez FJ was the most prolific author (17 articles). The USA and University of Murcia were the leading country and institution in this field with 219 and 22 publications, respectively. There were active collaborations among the authors, countries, and institutions. Hot topics focused on the interaction between epilepsy and circadian rhythm, as well as possible novel treatments.

### Active Cooperation Is Urgent

Although the number of publications about epilepsy and circadian rhythm fluctuated each year, epilepsy and circadian rhythm have been proven to be a hot research field since their emergence. Active cooperation was observed between prolific authors and developed countries especially the USA and European countries. Besides, co-authors analysis and co-cited references analysis suggested that high-frequency co-cited references did not come from prolific authors. Therefore, it is urgent for developing countries to encourage institutions to participate in research, strengthen cooperation, promote the development of related fields, and publish high-quality articles.

### Hot Issues in Epilepsy and Circadian Rhythm Research

Keywords are the high-level summarization and conciseness of the topic of an article. During the analysis process, the commonly used keywords were often used to identify hot issues in a research field. Results of co-occurring keywords and cluster analysis suggested that major ongoing research trends include the circadian rhythm of epilepsy based on different epileptic focus and the interaction between epilepsy and circadian rhythm, especially through MT, sleep–wake cycles, and clock genes, which may implicate possible treatments (such as chronotherapy and neural stem cell transplantation) for epilepsy in the future.

#### Epileptic Focus

The circadian rhythm of epilepsy occurrence is influenced by the localization of epilepsy. After analyzing the clinical seizures of 170 consecutive epilepsy patients, Gurkas et al. (17) found that seizures in children occur in specific circadian patterns depending on seizure onset location: generalized seizures were seen most frequently in wakefulness; frontal lobe seizures were seen at night and in sleep. In children, temporal lobe seizures occurred more frequently in wakefulness, usually in early evening (9, 18, 19, 37). Spencer et al. (20) used the NeuroPace RNS System to record objective and long-term rhythmicity of epileptiform activity and found that long episodes and long episodes validated as electrographic seizures patterns varied by region. The above results suggest implications for future treatments based on different epileptic focus.

#### Type

The diurnal pattern of seizure occurrence is also affected by the type of epilepsy (generalized or focal). Studies showed that the occurrence of generalized seizures and focal seizures originating from the parietal lobe in particular followed the circadian rhythm of cortisol (21).

#### Children

Changed circadian rhythm is evident in children with epilepsy. The results of Loddenkemper et al. (9) showed that, in children with focal epilepsy, frontal lobe seizures occurred predominantly during sleep, while temporal lobe seizures happened mostly during wakefulness. Generalized epilepsy often happens during daytime when children are wake (17, 22). Passarelli et al. (23) found that young patients with epilepsy associated with unilateral mesial temporal sclerosis had more seizures than middle-aged/old patients between 16:01 and 20:00 during video-EEG monitoring. Whether age plays an important role in the occurrence of epilepsy needs further study.

#### Melatonin

The pineal gland is a major component of the mammalian circadian rhythmicity system, which synthesizes MT at night. The secretion of MT shows a regular pattern of less secretion during the day and more secretion in the night, white sputum, and low night, which is synchronized with the peripheral light–dark cycle (24), and has a wide range of physiological functions such as maintaining circadian rhythmicity, promoting sleep, and enhancing human immune function. In various animal models of epilepsy, MT plays an antiepileptic effect (25–27). Mevissen et al. (28) found that the threshold of epileptic release in amygdala-ignited model rats was significantly increased by about 200 or 250% after 30 min of intraperitoneal injection of MT (75 or 100 mg/kg), which may significantly reduce the sensitivity of seizures in rats. The increase in epilepsy caused by MT of 75 mg/kg is comparable to the increase in post-release caused by conventional antiepileptic drugs such as carbamazepine, valproate, and phenytoin, and MT can also prevent secondary epileptic seizures (29). Significant clinical improvement in seizure activity was reported during MT treatment, particularly during the night (30). However, this conclusion needs to be further verified due to conflicting results. A Cochrane review published by Brigo (31) concluded that it was impossible to confirm the role of MT in reducing seizure frequency or improving quality of life in people with epilepsy and that adverse events of MT were not systematically evaluated.

#### Clock Genes

Cluster #5 indicated the vital role of clock genes in the circadian variation of epileptic excitability in epilepsy. By performing transcriptome analysis on human epileptogenic tissue, Li et al. (32) reported that disruption of clock circadian regulator (CLOCK) altered cortical circuits and may lead to generation of focal epilepsy. The BMAL1 gene, which codes for the binding partner of CLOCK to form the transcription CLOCK–BMAL1 complex, is also directly involved in epilepsy as proven by animal research (33). Leite Goes Gitai et al. (34) reported that, for mesial temporal lobe epilepsy patients, the imbalance of core clock genes in the epileptic hippocampus may affect the phase and amplitude of different genes that play roles in the electrical activity of neurons with consequences on the rhythmic fluctuation in the inhibitory/excitatory imbalance. The results of Matos et al. (35) also showed that altered temporal expression of the clock genes after a post-status epilepticus model suggests that clock genes may be involved in epilepsy. Therefore, chronotherapy and neural stem cell transplantation, as well as light, diet, exercise, and other zeitgebers, could modulate the rhythms in the hippocampus, and related structures may bring clinical benefits for reducing epilepsy. However, these genes and transcription factors should be further studied except in patients with lesional epilepsy associated with focal cortical dysplasia and tuberous sclerosis complex (36).

#### Sleep–Wake Cycles

As one of the representatives of circadian rhythm, sleep and wakefulness are easy to observe, which dominate epilepsy time biology studies and are the most robust predictors of seizures (9, 37). Animal experiments further confirmed the relationship between epilepsy occurrence and circadian rhythm (38). It is reported that sleep–wake-related patterns may have a role in seizure susceptibility (39), especially in patients with frontal lobe epilepsy, where sleep-related seizures are more evident (40). Temporal lobe complex partial seizures decrease rapid eye movement (REM) sleep (41). and non-REM sleep could favor epilepsy occurrence (40, 42), which may be supported by topographical EEG analyses (43).

#### Subjects

Keywords with citation bursts showed that 3 keywords (seizure, children, and epilepsy) with citation bursts continue to 2020, indicating that children with epilepsy has been getting great attention in recent years. Future studies could apply the results so far to human research subjects, especially children.

### Strengths and Limitations

To our knowledge, this is the first study to use the co-occurrence and co-citation analysis methods by CiteSpace to perform bibliometric analysis and visual display of the epilepsy and circadian rhythm from hot spots, co-cited references, and cooperation among authors, countries, and institutions. However, our study still has some limitations. Limited by the CiteSpace software, we analyzed only English studies in WoS; therefore, the data may not be comprehensive enough, and our results may not be applied to research published in other languages. Besides, due to the existence of multiple synonyms, when clustering keywords, there may be some overlap between different categories of content.

## Conclusions

Based on the results of CiteSpace, the current study suggested active cooperation between authors, countries, and institutions. Major ongoing research trends include the circadian rhythm of epilepsy based on different epileptic focus and the interaction between epilepsy and circadian rhythm, especially through MT, sleep–wake cycles, and clock genes, which may implicate possible treatments (such as chronotherapy and neural stem cell transplantation) for epilepsy in the future.

## Author Contributions

YZ designed and analyzed the data. DZ and SL drafted the manuscript and edited the manuscript. LZ analyzed the data. RJ contributed to revising the manuscript. All authors contributed to the article and approved the submitted version.

## Conflict of Interest

The authors declare that the research was conducted in the absence of any commercial or financial relationships that could be construed as a potential conflict of interest.
